# Enhanced Thalamic Functional Connectivity with No fMRI Responses to Affected Forelimb Stimulation in Stroke-Recovered Rats

**DOI:** 10.3389/fncir.2016.00113

**Published:** 2017-01-10

**Authors:** Woo H. Shim, Ji-Yeon Suh, Jeong K. Kim, Jaeseung Jeong, Young R. Kim

**Affiliations:** ^1^Department of Radiology, ASAN Medical Center, University of Ulsan College of MedicineUlsan, South Korea; ^2^ASAN Institute for Life Sciences, ASAN Medical Center, University of Ulsan College of MedicineUlsan, South Korea; ^3^Department of Bio and Brain Engineering, Korea Advanced Institute of Science and TechnologyDaejeon, South Korea; ^4^Department of Radiology, Athinoula A. Martinos Center for Biomedical Imaging, Massachusetts General Hospital, BostonMA, USA

**Keywords:** stroke recovery, rat, resting state fMRI, stimulus-induced fMRI, plastic reorganization

## Abstract

Neurological recovery after stroke has been extensively investigated to provide better understanding of neurobiological mechanism, therapy, and patient management. Recent advances in neuroimaging techniques, particularly functional MRI (fMRI), have widely contributed to unravel the relationship between the altered neural function and stroke-affected brain areas. As results of previous investigations, the plastic reorganization and/or gradual restoration of the hemodynamic fMRI responses to neural stimuli have been suggested as relevant mechanisms underlying the stroke recovery process. However, divergent study results and modality-dependent outcomes have clouded the proper interpretation of variable fMRI signals. Here, we performed both evoked and resting state fMRI (rs-fMRI) to clarify the link between the fMRI phenotypes and post-stroke functional recovery. The experiments were designed to examine the altered neural activity within the contra-lesional hemisphere and other undamaged brain regions using rat models with large unilateral stroke, which despite the severe injury, exhibited nearly full recovery at ∼6 months after stroke. Surprisingly, both blood oxygenation level-dependent and blood volume-weighted (CBVw) fMRI activities elicited by electrical stimulation of the stroke-affected forelimb were completely absent, failing to reveal the neural origin of the behavioral recovery. In contrast, the functional connectivity maps showed highly robust rs-fMRI activity concentrated in the contra-lesional ventromedial nucleus of thalamus (VM). The negative finding in the stimuli-induced fMRI study using the popular rat middle cerebral artery model denotes weak association between the fMRI hemodynamic responses and neurological improvement. The results strongly caution the indiscreet interpretation of stroke-affected fMRI signals and demonstrate rs-fMRI as a complementary tool for efficiently characterizing stroke recovery.

## Introduction

Ischemic stroke impairs neurovascular and metabolic functions in the brain and causes neurologic disabilities. Although severely damaged neurons fail to regenerate at the cortical level, interestingly, lost or compromised sensorimotor functions often recover at later stages of stroke. One of the restorative mechanisms underlying such recovery has been linked with the brain plasticity, the brain’s ability to reconstruct neural pathways and synapses in response to the loss of function (Kalénine et al., 2010; Heiss and Kidwell, 2014; Furlan et al., 2015). Despite the high interest and recent efforts, it is as yet unclear whether (or how) the stroke-affected brain areas functionally reposition in unaffected regions and/or reform connections with other brain areas to compensate for the impaired functions.

For identifying brain regions associated with restorative processes, task/stimulus-induced functional MRI (fMRI) has been frequently used to visualize brain activities associated with the neurologic recovery (for review see references Macey et al., 2015; Tang et al., 2015). More recently, resting state fMRI (rs-fMRI) has also provided a platform to explore spatiotemporal changes in neural connection across a wide range of brain regions. In general, by exploiting the temporal correlation of blood oxygenation level-dependent (BOLD) fMRI signals, the rs-fMRI has become an important method to assess the *in vivo* neuro-network (Carter et al., 2012; Grefkes and Fink, 2014; Thiel and Vahdat, 2015). Previous rs-fMRI investigations have reported that post-stroke loss and recovery of functions were associated with deterioration and subsequent retrieval of functional connectivity in the neural system, especially the interhemispheric connectivity changes (van Meer et al., 2010, 2012; Park et al., 2011). Based on these findings, alterations in the functional fields identified by either evoked fMRI or neural connectivity have been linked with the post-stroke functional recovery.

Past fMRI observations have suggested that remaining brain tissue, particularly the augmented neural activity in the contra-laterally homologous regions likely accounts for the restored sensorimotor function after stroke (Carey et al., 2002; Calautti and Baron, 2003). Typically, the assumption of intact neurovascular coupling underpins the interpretation of altered fMRI signals (Dijkhuizen et al., 2001; Kim et al., 2005). However, this link was challenged by us using multi-faceted fMRI measurements, in which the BOLD/CBV response ratio was significantly smaller in the stroke rats compared to the normal controls (Kim et al., 2005, 2006). Moreover, unclear relationship between fMRI and neurological recovery (i.e., complete absence of fMRI responses corresponding to the behavioral recovery) and questionable baseline physiology (e.g., choice of anesthesia) confounded the clear understanding of previous study results. (Weber et al., 2008; van Meer et al., 2010, 2012) The current study was designed to compare the functional fields and signal amplitudes acquired from both evoked fMRI and rs-fMRI in the stroke rat models exhibiting nearly full neurological recovery. Only using rats with large unilateral lesion encompassing most of the sensory and parts of the motor areas, the study focused on the role of sensorimotor activities in the contra-lesional hemisphere.

We hypothesized that in the chronic phase of stroke recovery, reinforced neural connections among the remaining intact brain regions are utilized more than the simple functional replacement and/or expansion of evoked activation toward the contra-lesional hemisphere. A well-established fMRI protocol with electrical stimulation of the rat forelimb was used to define the active sensorimotor brain regions (Dijkhuizen et al., 2003; Kim et al., 2005) while the BOLD rs-fMRI was used to investigate the functional connectivity networks. Noting that the proper brain function requires not only localized activation but also the integration of neural activities across multiple brain regions, the current study may elucidate the relationship between the different fMRI approaches to improve our understanding of the post-stroke recovery process and offer clues to the underlying neurobiological mechanisms.

## Materials and Methods

### Animal Surgery and Treatment

Using adult Sprague Dawley rats (Charles River, Wilmington, MA, USA, *n* = 12, 250 to 280 g), temporary stroke was induced with 2 h-occlusion of the right middle cerebral artery (MCAO) by advancing an intraluminal filament up into the internal carotid artery. Experimental protocols were approved by the Institutional Subcommittee on Research Animal Care, in accordance with the National Institutes of Health Guide for the Care and Use of Laboratory Animals. At around 6 months after the temporary MCAO, MRI experiments were performed. Age-matched normal rats were used as control group (*n* = 11).

Before positioning rats in the MRI machine, the animals were initially anesthetized with 1.5% isoflurane in a mixture of O_2_ and N_2_ gases (3:7). Polyethylene catheters were inserted into both left and right femoral veins for infusion of anesthetics and contrast agent administration, respectively, and also into the right femoral artery for monitoring of arterial blood pressure. Prior to the MRI experiment, isoflurane was disconnected and replaced by a continuous infusion of alpha-chloralose (∼30 mg/kg/h) with pancuronium (∼1.25 mg/kg/h), preceded by a loading bolus of ∼20 and ∼1.0 mg/kg of alpha-chloralose and pancuronium, respectively. Body temperature was maintained by a water-circulated heating pad, during which blood oxygen saturation, blood pressure and heart rate were continuously monitored throughout the MRI experiments. Each rat was mechanically ventilated at a rate of 40 strokes per min throughout the experiment with a 1:1 mixture of O2 and room air.

### Neurological Scoring

At 1, 3, 7, 11, 14, 60, and 180 day(s) after onset of stroke, the neurological status of each rat was evaluated using a modified grading system based on those previously described (Kim et al., 2005; van Meer et al., 2010). Total neurological score was a composite of motor (muscle status, abnormal movement), sensory (tactile and proprioceptive) and reflex tests. The sum of partial scores gave the total neurological score, which is graded on a scale of 0 to -20 points (normal score 0; maximal deficit score -20).

### Magnetic Resonance Imaging Data Acquisition

Magnetic resonance images were acquired on a horizontal bore 9.4T Bruker/Magnex system equipped with a home-built head surface RF transmit and receive coil with an approximate diameter of 3 cm. Multi-slice T2^∗^ maps were created by conventional multi-echo gradient-echo pulse sequences where TR/TE = 1000/[4, 7, 10 and 13] ms, from which lesion volumes were calculated using the image analysis software AFNI (Cox and Hyde, 1997). The lesion was defined as the ipsi-lateral parenchymal brain areas with T2^∗^ values higher than the average +2 standard deviations (s.d.) of the T2^∗^ values in contra-lateral tissue. For further analysis, apparent diffusion coefficient (ADC) maps were created with a diffusion-weighted EPI pulse sequence with TR/TE = 3700/40 ms and *b* = [5, 300, 800, and 1200] s/mm^2^. To obtain fractional anisotropy (FA), diffusion tensor imaging was performed with the acquisition of a reference image (*b* = 0) and six gradient directions, with a total *b* = 1200 s/mm^2^ in each direction.

### Stimulus Induced fMRI Acquisition

Thin copper wires were subcutaneously inserted into both forepaws at the wrist for electrical stimulation. A constant current generator was used, in which the threshold for stimulation was determined by detecting the onset of muscle flexion. To ensure supermaximal stimulation, the applied current during fMRI was approximately 0.2 mA higher than the threshold, which ranged from 1 to 1.2 mA. The stimulus duration and frequency were 0.3 ms and 3 Hz, respectively.

The fMRI activations acquired via BOLD and cerebral blood volume weighted (CBVw) techniques were measured approximately 1 h after the discontinuation of isoflurane (Gradient Echo Planar Imaging, TR/TE = 3700/15 ms for BOLD, TR/TE = 3700/11 ms for CBVw; FOV = 2.5 cm × 2.5 cm, nine contiguous 1 mm slices, and 64 × 64 matrix). A unilateral electrical stimulation paradigm, consisting of three periods of 37 s ‘stimulation on’ separated by 185 s ‘stimulation off,’ was alternated between the left and right forepaw and was repeated 3 to 5 times each for BOLD and CBVw methods. After the BOLD fMRI acquisitions, the blood pool contrast agent (MION) was intravenously administered (36 mg FeO2/kg), and the same stimulation paradigm was repeated.

### Resting State fMRI Acquisition

The rs-fMRI data (Gradient Echo Planar Imaging, TR/TE = 1000/12.89, FOV = 2.5 cm × 2.5 cm; nine contiguous 1 mm slices, and 64 × 64 matrix) were collected for 10 min (600 time points) before the administration of MION.

### Data Analysis

For the analysis of evoked fMRI data, rat images from each session were aligned to a template using nine degrees of freedom (three translations, three rotations, and three inflations). And multiple runs within each session were averaged into a single paradigm consisting of three stimulus/rest epochs for each rat; thereafter, BOLD or CBV data were concatenated across animals for detecting group functional activations as described in previous studies (Kim et al., 2005, 2007b, 2008). Data were analyzed using the standard general linear model approach (Friston et al., 1995), in which the stimulus paradigm is convolved with the respective hemodynamic response functions for the BOLD or CBV response to generate a maximum likelihood estimator. Functional activation maps were computed voxel by voxel using an equivalent *t*-test between the on and off stimulus periods. Unless specified otherwise, the statistical threshold for significant activation response was *p* < 0.0001 with a Bonferroni correction for multiple comparisons throughout the measured brain volume (Kim et al., 2005).

For rs-fMRI data, regions of interest (ROIs) were drawn freehand, using AFNI software based on the rat brain atlas defined by Paxinos and Watson (1997). Four brain regions associated with the sensorimotor function were chosen as seed ROI’s for analyses; contra-lesional M1/M2, contra-lesional S1fl, contra-lesional thalamus (TA) and ipsi-lesional TA. The time series from motion-corrected images for each voxel were detrended to the second order and bandpass-filtered between 0.01 and 0.3 Hz using AFNI software (Cox, 1996). Afterward, spatial smoothing (with a 0.5 mm full width at half-maximum Gaussian kernel) was applied on the rs-fMRI time series. Functional connectivity was assessed using the correlation coefficient value between the averaged time course from seed-ROI’s and each individual voxel’s time course. For variance stabilization, *r* was Fisher-transformed according to *z* = ln [(1 + *r*) / (1 - *r*)] / 2. Whole-brain functional connectivity maps (fc-maps) were obtained by voxel-wise calculation of *z* with the mean time series from a seed region as reference. After we co-registered all images to the rat brain atlas using AFNI (we provided the rat brain atlas to AFNI group, named mgh_wh_templete), group fc-maps were obtained by averaging across subjects. The group difference between normal and stroke-recovered rats was calculated using unpaired, two-tailed *t*-test. The resulting t-maps were then thresholded at the corrected *p* < 0.05 via Monte Carlo simulation with 10,000 iterations (AlphaSim, AFNI; minimum cluster size was set to 24 voxels). All data analyses were processed using MATLAB (Mathworks, Natick, MA, USA) and AFNI.

## Results

### Stroke Lesion Size and Location

**FIGURE 1:**
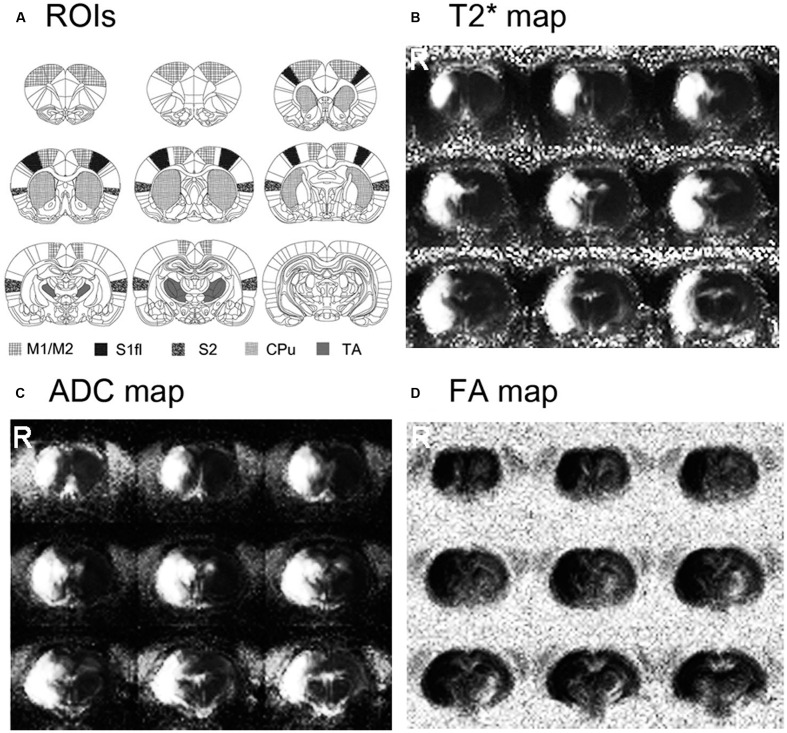
**Stroke location and size are shown on the averaged T2^∗^, apparent diffusion coefficient (ADC) and fractional anisotropy (FA) maps of stroke-recovered rats.** Stroke affected most of the sensorimotor-related regions in the right hemisphere, including M1/M2, S1, S2, caudate–putamen (CPu) and parts of thalamus (TA).

Stroke size and location are shown in **Figure 1**. The rats that underwent MCAO surgery showed severe tissue damage, confirmed by both diffusion- and T2-weighted MR images in most of the sensorimotor related regions in the right hemisphere, including the motor cortex (M1/M2), primary sensory cortex (S1), secondary sensory cortex (S2), caudate–putamen (CPu) and some parts of the TA. The lesion volumes, normalized by the contra-lesional volume, were 40.08 ± 5.7% of the contra-lesional hemisphere volume (109.6 ± 16.44 mm^3^).

### Neurological Scoring

**FIGURE 2:**
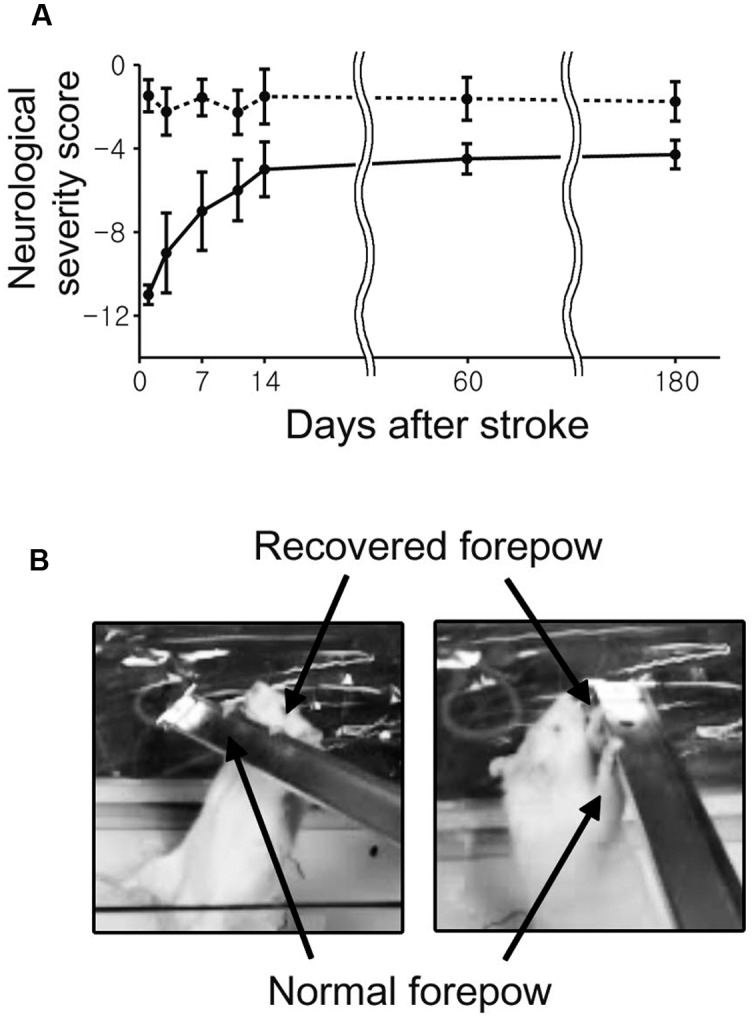
**The average neurological scores as a function of time after middle cerebral artery (MCAO) are shown with standard error.** **(A)** The neurological status of each rat was evaluated at 1, 3, 7, 11, 14, 60, and 180 day(s) after the onset of stroke. **(B)** All rats were able to use the impaired forelimb to hang on a bar after 180 days.

The stroke group showed spontaneous and gradually increasing recovery of sensorimotor functions. Relatively rapid behavioral improvement was observed over the early period from 1 to 14 days compared to the relatively slow recovery between 14 to 60 days (**Figure 2**). Although severe neurological deficits were observed immediately after the transient ischemia, most of stroke rats displayed almost fully recovered sensorimotor performances at days 60 and 180.

### Stimulus-Induced fMRI

**FIGURE 3:**
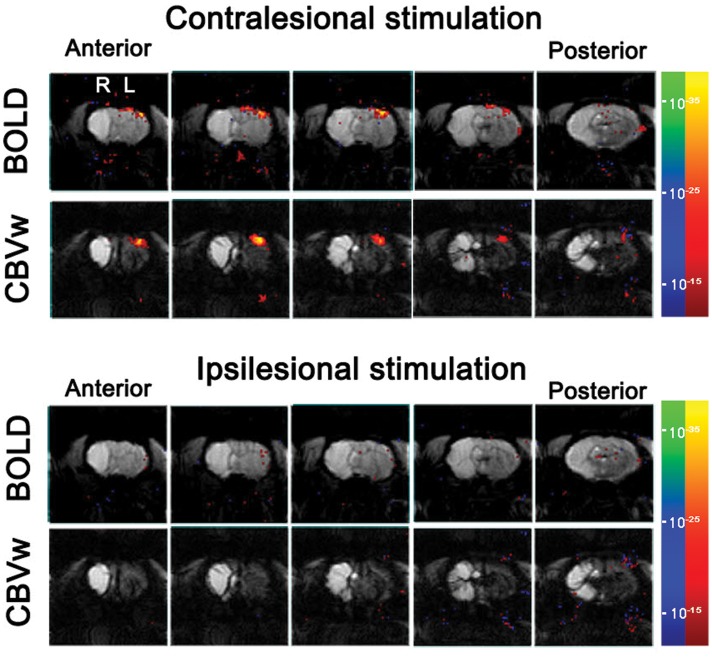
**Stimulus-induce functional MRI response maps of a representative stroke-recovered rat after 60 days of MCAO, acquired by stimulating unaffected (contra-lesional stimulation) and affected (ipsi-lesional stimulation) forelimbs using blood oxygenation level-dependent (BOLD) and cerebral blood volume weighted (CBVw) methods (top and bottom rows, respectively), with the activation threshold of *p* < 10^-15^.** The continuous five slices across primary-sensory cortex are shown.

**FIGURE 4:**
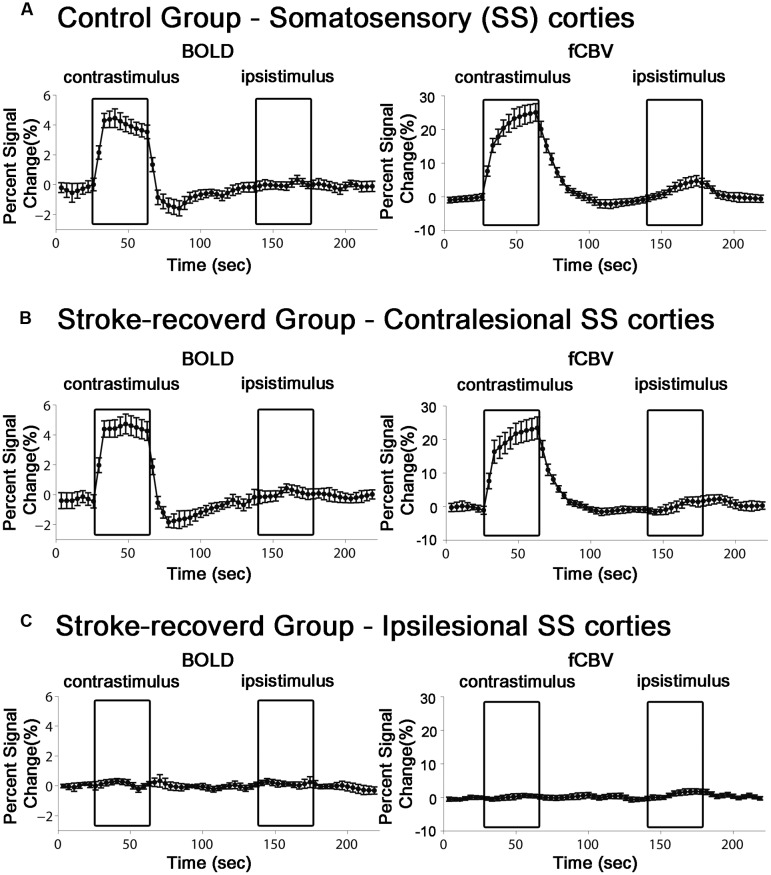
**Average time course of the fMRI percent signal change in both control (*n* = 11) and stroke-recovered animals (*n* = 12) for BOLD and CBVw responses.** ROIs were placed over the primary sensory cortex of forelimb (S1fl). Each contrastimulus or ipsistimulus was applied alternatively.

For all the unaffected cortices in the control group and contra-lesional hemispheres of the stroke group, robust fMRI activation was observed in the motor and forelimb sensory cortex (M1/M2 and S1fl, see **Figure 3**) during contra-lateral forelimb stimulation (i.e., contrastimulus) for both BOLD (increased signal) and CBVw (decreased signal, i.e., increase in CBV) methods (**Figure 4**). The center of activated regions was 0.1 mm anterior, 3.9 mm lateral, and 1.7 mm ventral to bregma (averaged over all stroke + normal animals), corresponding to S1fl according to Paxinos and Watson (1997). The difference in contra-lesional fMRI response magnitude was negligible between the two animal groups (unpaired, two tailed *t*-test). Upon stimulation of the unaffected forelimb, the fMRI time courses revealed a maximum ∼4% signal increase and ∼25% decrease for BOLD and CBVw signals in the contra-lesional sensorimotor cortex, respectively. In particular, for CBVw fMRI responses, a significantly delayed (almost 11 s) ipsistimulus response (ipsi-lateral activity to unilateral stimulus: maximum 6% CBV increase) was observed whereas the contrastimulus activation onset was immediate (see right panel in **Figure 4A**). Both BOLD and CBVw signals observed in damaged cortices (in ipsi-lesional hemispheres of stroke group) showed little response to the electrical stimulation of the affected forelimbs (**Figure 3**). In general, the time courses from the intact ipsi-lesional sensorimotor regions indicate that there were no appreciable and immediate signal changes elicited by either contra-lateral or ipsi-lateral stimuli. Additionally, we observed a small but appreciable ipsistimulus CBVw response (∼3% CBV increases), which was highly delayed from the stimulus onset (see right panel in **Figure 4B**) in the unaffected hemispheres of both animal groups.

### Resting State fMRI

**FIGURE 5:**
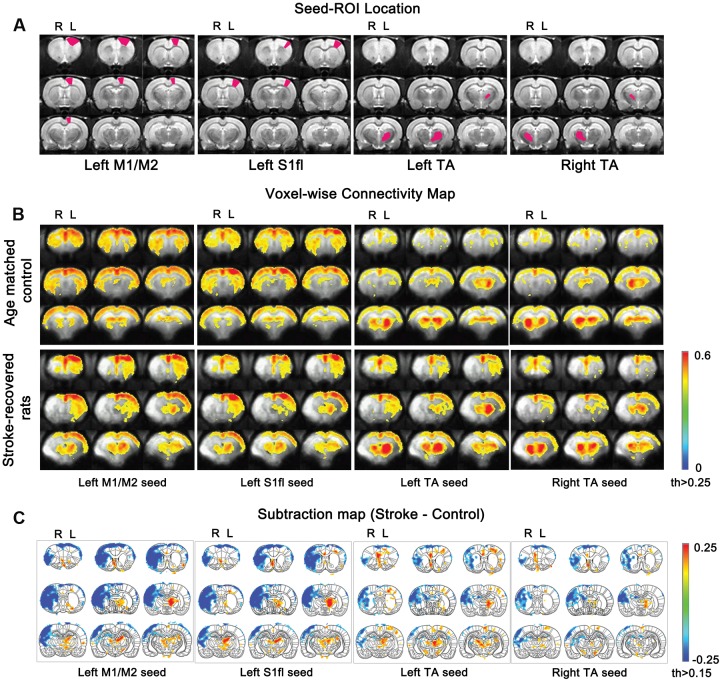
**Functional connectivity maps according to seed ROIs (M1/M2, S1fl and TA).** **(A)** seed ROI locations for each column. **(B)** Average connectivity values for all stroke-recovered rats are displayed with Fisher-transformed correlation coefficients (*z*) ranging from 0 to 0.6 with thresholds 0.25. **(C)** Subtraction maps [stroke-recovered minus age-matched control connectivity maps from **(B)**] are shown with *z* values (threshold > 0.15) over the rat brain atlas defined by Paxinos and Watson (1997).

To reveal the compromised functional connectivity network of post-stroke rats, four seed ROI’s were selected from the sensorimotor related regions; contra-lesional M1/M2, contra-lesional S1fl, contra-lesional TA and ipsi-lesional TA (**Figure 5**). The cross-correlation fc-maps were constructed using the low-frequency filtered BOLD signals (<0.15 Hz).

In control rats, we established the baseline fc-maps which demonstrated significant interhemispheric correlations between bilateral sensorimotor regions as previously described (Pawela et al., 2008; Zhao et al., 2008). Time courses from all four unilateral seed ROI’s were interhemispherically correlated between each other, particularly with those from the homologous counter parts in the opposite hemisphere (see upper panel in **Figure 5B**). The correlation using M1/M2 and S1fl seeds showed positive connectivity stretched over the entire contra-lateral cortex as well as the rest of ipsi-lateral cortex. Both the unilateral M1/M2 and S1fl seeds resulted in nearly equal connectivity to both ipsi-lateral and contra-lateral thalamus regions. Although the correlation map appeared slightly asymmetrical, the seed time course from the unilateral thalamus strongly correlated with the contra-lateral counterpart. Additionally, for the unilateral thalamus seed, a relatively weak but significant correlation pattern was found over the entire bilateral cortices (see upper panel in **Figure 5B**), which was nearly symmetrical and bilaterally equivalent in amplitude.

In stroke rats, both interhemispheric and intrahemispheric functional connections were absent within the liquefactive brain tissues in the ipsi-lesional hemisphere. In general, the analysis using both M1/M2 and S1fl seeds’ time courses revealed the correlation pattern (i.e., spatial distribution and strength of correlation) among sensorimotor regions in the contra-lesional hemisphere similar to those in control group. However, the relative correlation strengths between M1/M2 and S1fl were different, in which the M1/M2 seed time course resulted in a relatively higher correlation with S1fl region than that reciprocally acquired using the S1fl seed. For both M1/M2 and S1fl seed time courses, connections to the unaffected thalamic area were greater than those to the stroke-affected counterpart. Both ipsi- and contra-lesional thalamic seeds resulted in correlation patterns in the unaffected hemisphere, in which using the unaffected thalamus seed ipsi-laterally produced more spatially expanded and higher correlation than using the affected thalamus seed.

**FIGURE 6:**
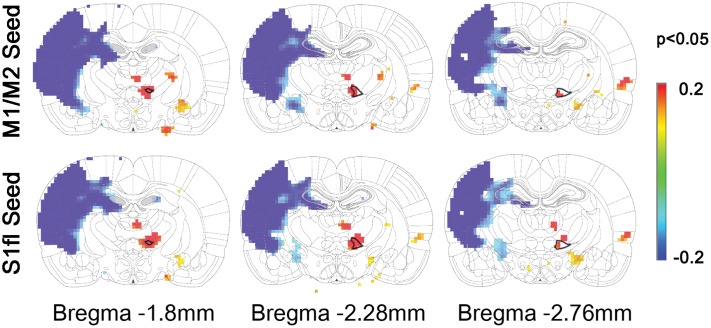
**The locations of significant difference between control and stroke-recovered function connectivity maps (the corrected *p* < 0.05 via Monte Carlo simulation) are shown on the subtraction maps.** The ventromedial thalamus (VM) was drawn with bold black line on the rat brain atlas in background.

**FIGURE 7:**
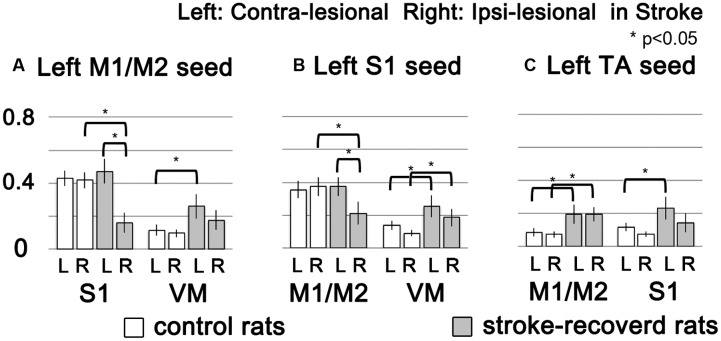
**The functional connectivity strength of target brain regions (M1/M2, S1 and VM) were showed in bar graphs according to seed ROIs (left M1/M2, left S1 and left TA) with standard error.** White and gray bars represent control and stroke-recovered rats, respectively (^∗^*p* < 0.05, unpaired between two groups and paired within a group, two-tailed *t*-test).

To demonstrate the group difference, subtraction of the correlation maps (stroke group minus control group) was obtained (**Figure 5C**). First, considerable spatial expansion of the overall functional connectivity was observed mostly in the contra-lesional hemisphere, particularly at the subcortical level. However, almost no enhancement of either correlation area or strength was observed in the ipsi-lesional subcortex. For both M1/M2 and S1fl seeds, the correlation values were significantly enhanced in the thalamus (the corrected *p* < 0.05 via Monte Carlo simulation using AlphaSim), particularly at the ventromedial thalamic nucleus (VM; **Figure 6**). These two seeds produced nearly identical, statistically significant stroke-enhanced activity patterns in the contra-lesional subcortex whereas the ipsi-lesional enhancement appeared minimal (**Figure 5C** first two panels from left and **Figure 6**). As shown in **Figures 7A,B**, the correlation coefficients also revealed significantly greater functional connectivity between VM and either one of two cortical seeds especially in the contra-lesional hemisphere. When the unaffected TA seed was used, the contra-lesional enhancement was also observed in cortical areas, where its functional connectivity with both M1/M2 and S1fl regions significantly increased as demonstrated in the subtraction map and correlation coefficient values (**Figure 5C** third panel from left and **Figure 7C**).

## Discussion

In this study, we analyzed both stimulus-induced and rs-fMRI data to investigate neuronal changes associated with stroke recovery. Specifically, the stimulus-induced fMRI was performed to assess the spatial reorganization of the localized brain activity while rs-fMRI was used to investigate altered neural networks among the intact brain regions. In particular, in order to emphasize the recovery-associated changes of neural activity in the remaining brain tissue, the experiments focused on the rat stroke model displaying nearly full neurological recovery despite the severe unilateral damage. Our primary hypothesis was to test whether the contra-lesional activity in response to impaired limb stimulation is significantly altered during the spontaneous post-stroke functional recovery, in which the ipsi-lesional hemisphere minimally participated.

As an effective means to explore post-stroke brain activity en masse, details of fMRI responses have been considered important and examined to provide clues for understanding functional recovery and related neural mechanisms (Tang et al., 2015; Thiel and Vahdat, 2015). In this regard, both experimental models as well as human patients have been used to investigate whether and how any spatiotemporal deviants in the fMRI activation pattern are associated with neurological and functional recovery and reformation of the damaged brain (Calautti and Baron, 2003; Kim et al., 2005). Altered BOLD responses and a spatial shift of fMRI activation have been reported, which imply the recovery-related modifications of stimuli-induced neural activities (Carey et al., 2002; Heiss and Kidwell, 2014). In particular, Dijkhuizen et al. (2001, 2003) reported strong presence of such plastic fMRI response in the contra-lesional hemisphere at acute/sub-acute stages of experimental stroke. The study showed spatially unfocused, widespread ipsistimulus CBVw fMRI activations in the contra-lesional hemisphere, which were induced by the stimulation of stroke-affected limbs of rats with a unilateral damage in both cortical and subcortical areas (Dijkhuizen et al., 2001, 2003). In contrast to these results, any bona fide fMRI activity in response to electrical stimulation of impaired forelimbs was not observed in the current study (**Figure 3**). In this particular stroke model with a very large ischemic infarct (**Figure 1**), thereby grossly limiting the activity in the ipsi-lesional hemisphere, surprisingly neither the intact sensorimotor regions in the contra-lesional hemisphere nor any other brain regions displayed stimuli-associated BOLD or CBVw fMRI activities.

The disagreement between these animal studies is likely due to the misinterpretation of artifactual signals of non-neural origin. In fact, we have shown a non-specific ∼20% increase of CBVw signal even in normal rats upon unilateral electrical forelimb stimulation (Kim et al., 2005), which was not confined to the sensorimotor areas (i.e., affecting the entire neocortex) and occurred throughout the entire bilateral cortices. In particular, this CBV rise only manifested with an ∼11 s temporal delay after the stimulus onset, resulting in the contra-lesional activation pattern, both temporally and regionally similar to that reported by Dijkhuizen et al. (2001). Further investigations also revealed that such temporally unsynchronized CBV surge did not accompany either significant BOLD or metabolic demand (i.e., Cerebral Metabolic Rate of Oxygen: CMRO_2_), validating the non-neural nature of the phenomenon (Kim et al., 2005). Additionally, Dijkhuizen et al. (2001, 2003) also reported mostly absent contra-lesional ipsistimulus fMRI responses at 14 days after stroke. These responses were later replaced by reinstatement of the activations in the ipsi-lesional hemisphere by the stimulation of stroke-affected limbs (Dijkhuizen et al., 2001, 2003). On the contrary, our current study, performed at more than 6 months after the transient MCAO, exhibited traces of the ipsistimulus cortical fCBV responses in both the stroke and control groups (**Figure 4**). Therefore, such delayed non-specific cortical CBV responses are not related to either the age of rat or the recovery status although no direct physiological basis of this CBVw signal has yet been ascertained.

Several past studies using rs-fMRI or stimuli-induced fMRI with electrophysiology reported the restoration of ipsi-lesional activities in correlation with the sensorimotor function recovery (Weber et al., 2008; van Meer et al., 2010). Interestingly, our current study using both BOLD and CBVw fMRI in animal models with very large stroke demonstrated that the dramatic neurological recovery did not accompany either contrastimulus fMRI activities (i.e., BOLD and CBV) in the ipsi-lesional hemisphere or any fMRI responses at all in the entire brain (**Figure 4**). This particular result was puzzling since both motor and sensory functions were behaviorally restored in the affected forelimbs of subjected animals. Therefore, the discrepancy strongly suggests a questionable mechanistic link between ipsistimulus fMRI activation in the contra-lesional hemisphere (via stimulation of the affected forelimb) and functional recovery. Similarly, such a complete absence of plastic and/or reorganized hemodynamic fMRI activity in the ipsi-lesional hemisphere was also previously observed and longitudinally validated by Weber et al. (2008) using both BOLD fMRI and electrophysiology. Although choice of anesthesia was discussed as a possible source of the suppression of plastic response by Weber et al. (2008) who used medetomidine (vs. alpha-chloralose by Dijkhuizen et al.) (Tsurugizawa et al., 2010; Liu et al., 2012), the prolonged lack of neural signals that should account for the apparent neurological recovery was neither questioned nor discussed in their study. Even our present scrutiny on this issue using both BOLD and CBVw fMRI techniques in the behaviorally recovered rats under the same anesthesia regimen previously used by Dijkhuizen et al. did not reveal any relevant fMRI signals.

These observations undermine the conclusions derived from other previous fMRI studies, which are based on the assumption of normal neurovascular coupling and/or recruitment of remaining intact brain regions that are relevant to restored functions. The compromised neurovascular coupling could in fact, bring about misleading interpretations of the fMRI signals and conclusions unrelated to the actual neural activities that are associated with stroke recovery. In line with the probable stroke-affected alteration of neurovascular coupling, we previously demonstrated abnormal fMRI hemodynamic characteristics during the recovery phase of stroke, in which the BOLD fMRI response was significantly diminished while the CBVw response was nearly restored to the normal level. Therefore, prior knowledge of such a mismatch is highly important before applying fMRI as correlating indices of neuronal activity. Alternatively, the stimulus-evoked change in local metabolism (i.e., stimulus-induced percent CMRO_2_ change in somatosensory area) has been suggested as a marker of functional recovery rather than the hemodynamic fMRI parameters alone (Kim et al., 2005, 2007a). In this regard, a multi-faceted fMRI approach (i.e., BOLD, CBVw, CBF) is necessary to prevent the inaccurate depiction of neural events in the stroke-affected brain.

Therefore, such complete lack of fMRI activity observed in the current study indicates possible dissociation of the hemodynamic fMRI response from the neurological recovery characteristics and also may imply an intrinsic limitation of the anesthetized rat stroke model for studying fMRI-based neural signals. The results warrant further investigation into a gamut of biophysical signals that solely reflect the neural activity in the entire central nervous system, and are not limited to the known sensorimotor areas and cerebrum. The effects of general anesthesia (vs. awake) should also be considered in the stroke recovery model studies. Although the exact neurophysiological mechanisms underlying stroke recovery are yet to be identified in these rat model studies, the current findings strongly caution the interpretation of stroke-affected fMRI signals without understanding the direct neural correlates.

Upon analysis of rs-fMRI data, the correlation maps in the contra-lesional cortex generated by using either M1/M2 or S1fl seed in stroke rats were nearly equal to those of the age-matched controls; almost no residual cortical correlation was observed in the subtraction maps (**Figure 5C**, first two panels from left). However, in the subcortex, the contra-lesional connectivity between M1/M2 and S1fl (as seeds), and thalamus was significantly elevated, particularly in the VM region (**Figures 5C**, **6**, and **7A,B**). The ventromedial nucleus is a well-recognized center of neural pathways involved with motor control, which is likely associated with the increase of spontaneous sensorimotor activity during stroke recovery (Desbois and Villanueva, 2001). Similarly, the contra-lesional thalamic seed also revealed significantly increased connectivity in both M1/M2 and S1fl, compared to the control results (**Figure 7C**), whereas the correlation map derived from the ipsi-lesional thalamus seed was similar to that found in the normal control (**Figure 5C** right most panel; the subtraction map shows almost no residual correlation). This particular result suggests a preferential accruement of thalamus-sensory resting state connectivity in the contra-lesional hemisphere during the recovery phase of stroke. Additionally, to examine the dependence of the rs-fMRI results on analysis strategy, we have performed the data analyses using global signal regression (GSR). Although the GSR-related noises were detected [**Supplementary Figure 1**, i.e., anti-correlation in the necrotic region (Schölvinck et al., 2010; Murphy et al., 2013; Pan et al., 2015)], the inclusion of GSR did not affect the general study outcome and also displayed the significant enhancement of the rs-fMRI connectivity in the VM region (**Supplementary Figure 2**). However, such reinforced thalamic connectivity and the lack of changed cortical rs-fMRI are in disagreement with previous study results obtained by van Meer et al. (2012) in large (cortical and subcortical) stroke models. The measurement method was similar; however, the choice of anesthesia was isoflurane (vs. alpha-chloralose in the current study). Since isoflurane is a potent vasodilator and known to decrease neural activity and interfere with spontaneous neural dynamics (e.g., burst suppression), it might reduce the detection power of rs-fMRI connectivity and alter the final outcomes at both vascular and neural levels (Williams et al., 2010). On the other hand, alpha-chloralose is one of the most widely used anesthetics in fMRI experiments in rodents and has been found to preserve the specific functional BOLD responses and functional connectivity patterns, compared with other anesthetic agents (Peeters et al., 2001; Majeed et al., 2009; Williams et al., 2010).

## Conclusion

Our study using the neurologically recovered rats from severe unilateral stroke revealed that the stimulus-induced fMRI alone is insufficient for characterizing stroke recovery. We suggest that the functional connectivity analysis using rs-fMRI can be used as a complementary tool since the intra-hemispheric functional connectivity in contra-lesional subcortex is highly involved in the stroke recovery process. The results demonstrate that the consolidation of intra-hemispheric functional connectivity among sensorimotor areas in the contra-lesional hemisphere may play a critical role in the recovery of somatosensory and motor functions after stroke.

## Author Contributions

Substantial contributions to the conception or design of the work; or the acquisition, analysis, or interpretation of data for the work; (WS, JJ, and YK). Drafting the work or revising it critically for important intellectual content; (J-YS, JK, and JJ). Final approval of the version to be published; (YK). Agreement to be accountable for all aspects of the work in ensuring that questions related to the accuracy or integrity of any part of the work are appropriately investigated and resolved; (WS, J-YS, JJ, and YK).

## Conflict of Interest Statement

The authors declare that the research was conducted in the absence of any commercial or financial relationships that could be construed as a potential conflict of interest.
