# Supplementary material for: Enhanced Thalamic Functional Connectivity with No fMRI Responses to Affected Forelimb Stimulation in Stroke-Recovered Rats
Source: Front Neural Circuits. 2017 Jan 10;10:113. doi: 10.3389/fncir.2016.00113 (PMC5222821; doi:10.3389/fncir.2016.00113)
Supplement: Supplementary file 1 [file Data_Sheet_1.DOCX]

**SUPPLEMentary Figure LEGENDS**

**Supplement** **Figure 1.** Representative comparison between the analyses with and without GSR in a single stroke rat: (a) T2* weighted EPI image, corresponding (b) FA and (c) ADC map. Correlation maps obtained using the S1fl seed, analyzed with (d) and without (e) the GSR step. With GSR, the negatively correlated voxels are apparent in the necrotic region where the average correlation coefficient is ~ -0.35 whereas little correlation is observed in the same region without GSR.

**Supplement** **Figure 2.** Comparison of analyses (a) with and (b) without GSR in the statistically significant correlation coefficient difference maps between stroke and control groups: the contra-lesional VM area exhibits the significantly enhanced rs-fMRI compared to the age-matched controls (even with GSR).
