# Supplementary figures and images for: Enhanced Thalamic Functional Connectivity with No fMRI Responses to Affected Forelimb Stimulation in Stroke-Recovered Rats
Source: Front Neural Circuits. 2017 Jan 10;10:113. doi: 10.3389/fncir.2016.00113 (PMC5222821; doi:10.3389/fncir.2016.00113)

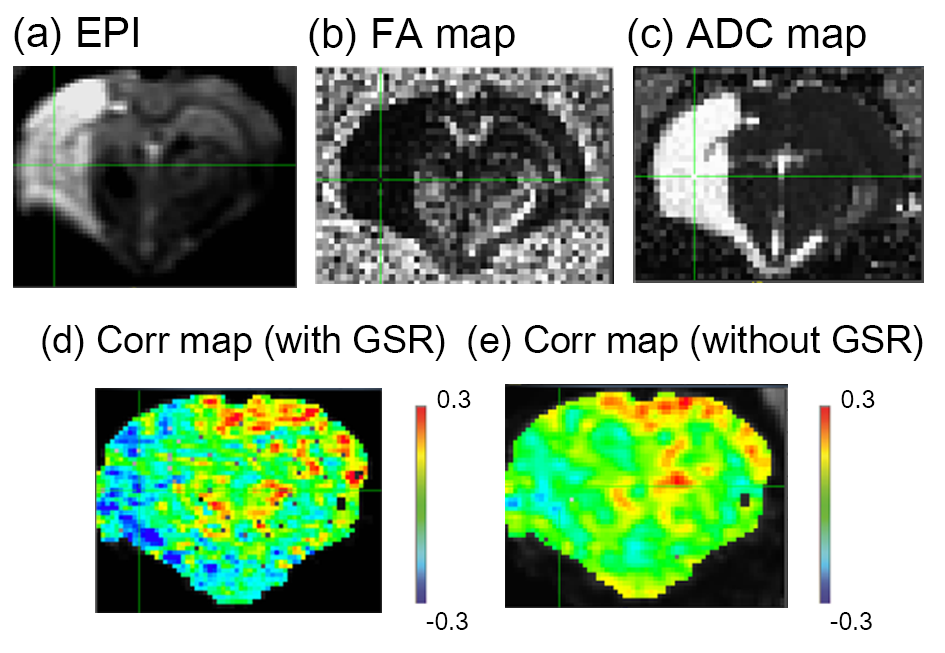

Supplement: Supplementary file 2 [file Image_1.TIF]

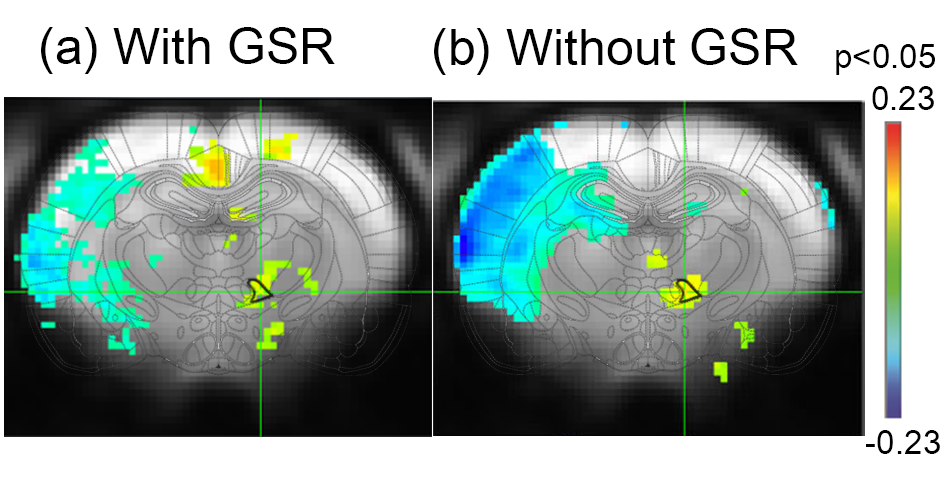

Supplement: Supplementary file 3 [file Image_2.TIF]
